# Preparation of Octadecyl Amine Grafted over Waste Rubber Powder (ODA-WRP) and Properties of Its Incorporation in SBS-Modified Asphalt

**DOI:** 10.3390/polym11040665

**Published:** 2019-04-11

**Authors:** Meizhao Han, Xiang Zeng, Yaseen Muhammad, Jing Li, Jing Yang, Song Yang, Yunhao Wei, Fei Meng

**Affiliations:** 1 School of Chemistry and Chemical Engineering, Guangxi University, Nanning 530004, China; hmzsourire@163.com (M.H.); zengxiang0718@163.com (X.Z.); Betsy0828@163.com (J.Y.); qq314593113@163.com (S.Y.); weiyunhaohg@163.com (Y.W.); 17862186993@163.com (F.M.); 2 Guangxi Key Laboratory of Petrochemical Resource Processing and Process Intensification Technology, Guangxi University, Nanning 530004, China; myyousafzai@gmail.com; 3 Institute of Chemical Sciences, University of Peshawar, Peshawar 25120, Pakistan

**Keywords:** waste rubber powder, covalent grafting reaction, mechanical properties, lipophilicity, composite network structure

## Abstract

Through a covalent grafting reaction, octadecyl amine (ODA) was grafted on the surface of waste rubber powder (WRP) to obtain an ODA-WRP modifier, which was in turn compounded with a styrene-butadiene-styrene block copolymer (SBS) to prepare ODA-WRP/SBS-modified asphalt. The three major indicators (i.e., dynamic shear rheometer (DSR), multi-stress creep recovery (MSCR), and separation tests) showed that 1-ODA-WRP effectively improved the complex shear modulus (G*), elastic Modulus (G′), and loss modulus (G″) by 36.47%, 40.57%, and 34.77% (64 °C and 10 Hz), respectively, as compared to pristine SBS-modified asphalt. Fluorescence microscopy (FM) results concluded that the enhancement in mechanical properties was accredited to the better compatibility of various components in asphalt and establishment of network structure between ODA-WRP and SBS in ODA-WRP/SBS-modified asphalt. Fourier infrared spectroscopy (FT-IR) and scanning electron microscope (SEM) analyses confirmed the successful synthesis of ODA-WRP. This study could be of great help in synthesizing ODA-WRP asphalt modified with SBS for highways and construction applications.

## 1. Introduction

Styrene-butadiene-styrene block copolymer (SBS)-modified asphalt is widely used in the preparation of asphalt-based roads because of its excellent mechanical properties and high service life [1,2,3]. However, with the development of the global automotive industry, the number of cars has increased rapidly, which has resulted in a corresponding increase in traffic pressure. With this increase, SBS-modified asphalt-based pavements have also raised some problems, such as rutting, cracking, and a shortened road life [4,5,6]. Many studies have been performed to improve the performance of SBS-modified asphalt via polymer composite modification [7,8,9,10], biomass material modification [11,12], and nanomaterial modification [13,14,15,16,17].

Development of the automotive industry has also increased the amount of waste rubber tires [18,19]. At present, disposal methods for used tires include incineration, recycling, and production of waste rubber powder (WRP) [20,21]. Among these, converting to rubber power (RP) has become the main treatment method of waste rubber tires because of its large output, low cost, and environmentally benign nature in the production process. However, these large quantities of wasted RP lack effective recycling methods. Among the many methods of application of WRP is its application in the preparation of modified asphalt. Jeong et al. [22] found that the viscosity and high temperature properties of WRP-added asphalt were considerably higher than pristine asphalt. Fang et al. [23] reported that the addition of WRP can improve the anti-aging performance of asphalt and prolong the service life of asphalt pavement. These results indicate that WRP-modified asphalt is feasible and provides an effective way for the disposal of waste rubber powder, though it needs to meet certain standards implemented in various countries [24,25]. However, Navarro et al. [26] found that the storage stability of rubber asphalt decreased with increasing particle size and storage temperature. This is because WRP is inert [27,28] and, hence, caused serious segregation problems in asphalt. In order to tackle this issue, researchers are working on the modification of WRP [29] with various approaches including activation treatment [30,31], modified grafting, etc. [32,33]. In our previous studies, octadecyl amine (ODA)-grafted graphene nanoplatelets (GNPs) were reported with excellent compatibility in asphalt [34]. In line with those studies, it is worth studying the effect of ODA as a modifier in WRP and, in turn, apply it in the preparation of modified asphalt.

Thus, herein, the compatibility of WRP in asphalt and further improvements of the properties of SBS-modified asphalt were carried out by grafting ODA via a covalent bonding grafting strategy [34]. ODA was decorated on the surface of WRP and was used as a modifier to prepare ODA-WRP/SBS-modified asphalt. The plasticity, high temperature stability, viscoelastic properties (using three major indicators and dynamic shear rheometer (DSR) test), compatibility (using segregation experiments), and rutting properties (using multi-stress creep recovery (MSCR) test) of ODA-WRP/SBS-modified asphalt with respect to different grafting amounts of ODA-WRP were analyzed. The existence of WRP in SBS-modified asphalt before and after modification was analyzed by fluorescence microscope (FM). The chemical structural changes and surface morphology of WRP before and after modification were analyzed by Fourier transform infra-red spectroscopy (FT-IR) and scanning electron microscopy (SEM).

## 2. Preparation of Octadecyl Amine Waste Rubber Powder/Styrene-Butadiene-Styrene (ODA-WRP/SBS) Composite Modified Asphalt

### 2.1. Materials

Base asphalt (SK-70A) was supplied by Xiamen Walter Group Co., Ltd., Xiamen, China. SBS block polymer (SBS, T6302H) was supplied by Dushanzi petrochemical branch, China National Petroleum Co., Ltd, Karamay, China. Absolute ethanol was obtained from CP, Guangdong chemical reagent engineering technology research, Guangzhou, China. 90% ODA was obtained from Shanghai Macklin Biochemical Co. Ltd, Shanghai, China. 

### 2.2. Preparation Method

#### 2.2.1. Preparation of ODA-WRP

**Figure 1:**
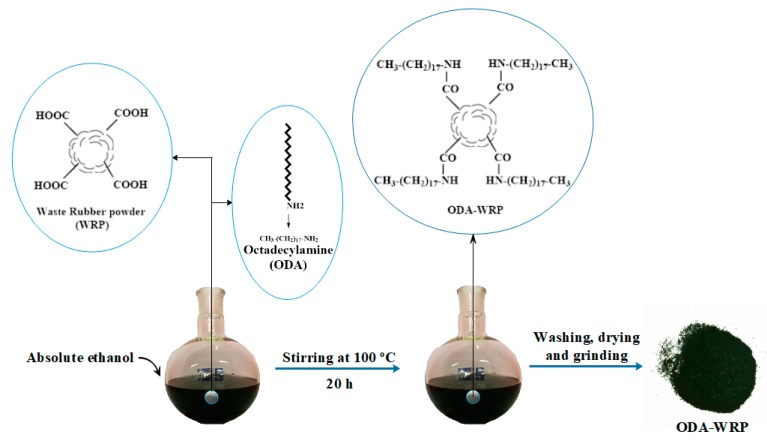
Preparation process of octadecyl amine grafted over waste rubber powder (ODA-WRP).

The preparation process of ODA-WRP is shown in Figure 1. After adding 20 g of the pre-washed WRP and 200 mL absolute ethanol to a 500 mL three-necked flask, ODA was added according to different ratios of WRP:ODA (20:2, 20:1, and 20:0.5) and stirred at 100 °C for 20 h. The product was then washed with suction, washed and filtered with absolute ethanol and deionized water, and dried at 60 °C in an oven to obtain ODA-WRP [34]. During the reaction, the amino group (–NH_2_) on the ODA reacted with the carboxyl group (–COOH) on the surface of WRP to form an amide (–NHCO–), thereby facilitating ODA grafting onto the WRP surface.

#### 2.2.2. Preparation of ODA-WRP/SBS Modified Asphalt

**Figure 2:**
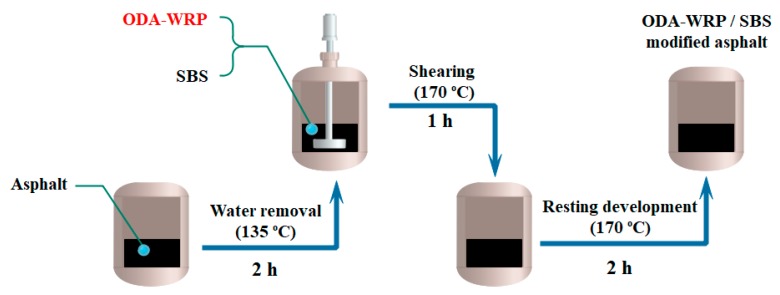
Preparation process of different kinds of WRP/styrene-butadiene-styrene (SBS)-modified asphalt.

Figure 2 schematically shows the preparation process of different kinds of WRP/SBS-modified asphalts, provided by Guangxi Transportation Research & Consulting Co., Ltd., China. All modified asphalts in this study were prepared via similar procedures. The amount of SBS and WRP added were 5% each, while the amount of ODA-WRP in different ratios was calculated based on the WRP content. First, the base asphalt was heated at 135 °C in an oven for 2 h to remove water, and then it was immediately stirred at 5000 rpm for 1 h to evenly disperse SBS and WRP. After that, the asphalt was heated at 170 °C in oven for 2 h and kept static for the complete development of asphalt. Compositions of different types of asphalts are shown in Table 1.

### 2.3. Analysis of Asphalts

Mechanical properties of ODA-WRP/SBS-modified asphalt, in terms of low temperature plasticity, stability, and viscosity, were determined by three major index tests (ductility, softening point, and penetration). The effect of WRP contents on the rheological properties of asphalt (before and after modification) were determined by DSR (Bohlin CVO). The high temperature stability of asphalt was determined with an MSCR (Bohlin CVO) test by studying the effect of WRP on the high temperature performance of asphalt samples.

WRP and three types of modified ODA-WRPs in asphalt were characterized by FM (IMAGER.Z2, Carl Zeiss, Jena, Germany). The change in surface functional groups of WRP before and after modification was analyzed by FT-IR (iS50, US NICOLET Co., Ltd., Mountain, WI, USA, and potassium bromide as a background). The elemental composition of the WRP surface before and after modification was analyzed by energy dispersive spectroscopy (EDS, Plano, TX, USA, oblique insert and flat insert spectrometer EDAX), while surface morphology of pristine and modified WRP was observed by SEM (SU8220, Hitachi, at an accelerating voltage of 5 KV). The Sputter Coaster (MSP-2S, IXRF, 8–10 pa of vacuum degree, 60 s of vacuum time, 15 s of coating time, and discharge current of 38 mA) was used to spray gold film (about 7.5 nm thickness) on the samples.

## 3. Results and Discussion

### 3.1. Analysis of Three Indicators

**Figure 3:**
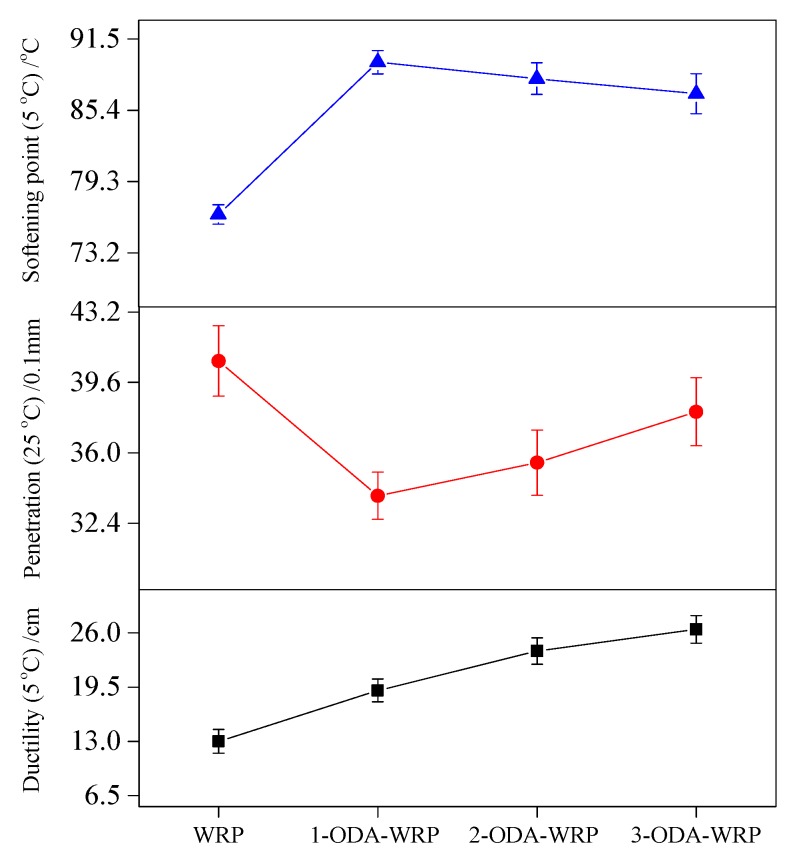
Effect of different WRPs on the performance of WRP/SBS-modified asphalt.

The three major indicators (ductility, softening point, and penetration) were used as the most basic and versatile test methods (JTG E20-2011 [35]) for analyzing asphalt performance. Ductility was generally tested at 5 °C and a tensile speed of 5 cm/min, while softening point followed the universal method at a starting test temperature of 5 °C. The penetration test was carried out with a standard needle for 5 s at a temperature of 25 °C. The samples used in each test were cured at different temperatures and variable lengths of time (i.e., for ductility, softening point, and penetration tests the curing times were 2, 1, and 2.5 h, respectively). The results of ductility, softening point, and penetration tests shown in Figure 3 suggest they had better values for ODA-WRP-modified asphalt than pristine WRP. 

Among the various samples, 3-ODA-WRP exhibited optimum effects on improving SBS asphalt ductility, penetration, and the softening point (i.e., an increase of 103.07% in ductility, 13.46% in the softening point, and 6.62% in penetration) as compared to that of unmodified WRP. Furthermore, with decreasing amounts of ODA grafted on the WRP surface, the ductility and penetration decreased, while the softening point increased. This could be related to the lipophilic group (–(CH_2_)_17_CH_3_) grafted on the surface of WRP, which can “catch and concentrate” the asphalt component near the WRP. The greater the grafting number of lipophilic groups (3-ODA-WRP), the greater are the chances of WRP catching asphalt components, and the greater the ductility of the asphalt will be. However, WRP can also absorb the light components (aromatics and saturated) in asphalt and, hence, swells and develops in asphalt [36,37]. Excessive lipophilic groups (higher WRP loading) will cause wrapping of WRP particles by heavy components (colloid and asphaltene) of asphalt, which will decrease the swelling rate of WRP. This assumption was further supported by the lower improvement of 3-ODA-WRP on the softening point and penetration. Upon comparing with literature reports, the modification effect of 5% ODA-WRP on SBS-modified asphalt on ductility, softening point, and penetration were better than some reports using high-content WRP or SBS-added asphalt [38,39,40], showcasing the superior nature of the proposed ODA-WRP modified asphalt.

### 3.2. Dynamic Shear Rheometer (DSR) Test of Various Asphalts

DSR is the primary instrument for viscoelastic studies specified by the United States Strategic Highway Research Program (SHRP). Through periodic stress or strain oscillations, the test can simulate changes in rheological properties of asphalt pavement by modeling vehicle conditions based on American Association of State Highway and Transportation Officials test standards (AASHTO 2013) [11,41,42]. The test results can collect data about various rheological properties of asphalt, like complex shear modulus G* and the phase angle δ. The essence of the complex shear modulus G* is the ratio of the sinusoidal alternating load stress to the strain, which characterizes the ability of the material to resist deformation under repeated shear forces. It proportionally shows the high temperature stability of the material. As a typical viscoelastic material, after being deformed by stress, the asphalt stores a part of its energy due to elasticity, and also consumes another part due to viscosity. G* is believed to be composed of two parts: viscosity and elasticity in the rheological properties of asphalt, and its relationship is shown by Equation (1) [15,43], where G′ is the storage modulus, which is directly proportionate to the elastic properties of asphalt. G″ is the loss modulus, and its value proportionally represents the viscous properties of asphalt. Both G′ and G″ can be calculated according to Equations 2 and 3, respectively [15,43], using G* and phase angle δ.

(1) $$G^{*}=G^{\prime }+G^{\prime \prime },$$

(2) $$G^{\prime }=\left|G^{*}\right|*cos\delta ,$$

(3) $$G^{\prime \prime }=\left|G^{*}\right|*sin\delta .$$

In this test, a frequency sweep range of 0.1–10 Hz was applied while varying test temperature (46, 52, 58, 64, 70, 76, and 82 °C). The stress was held at auto-stress, and the total strain was held at 12% to ensure rheological behavior of all asphalt binders in the linear viscoelastic range. The gap and the diameter were 1 and 25 mm, respectively.

#### 3.2.1. Complex Shear Modulus (G*)

**Figure 4:**
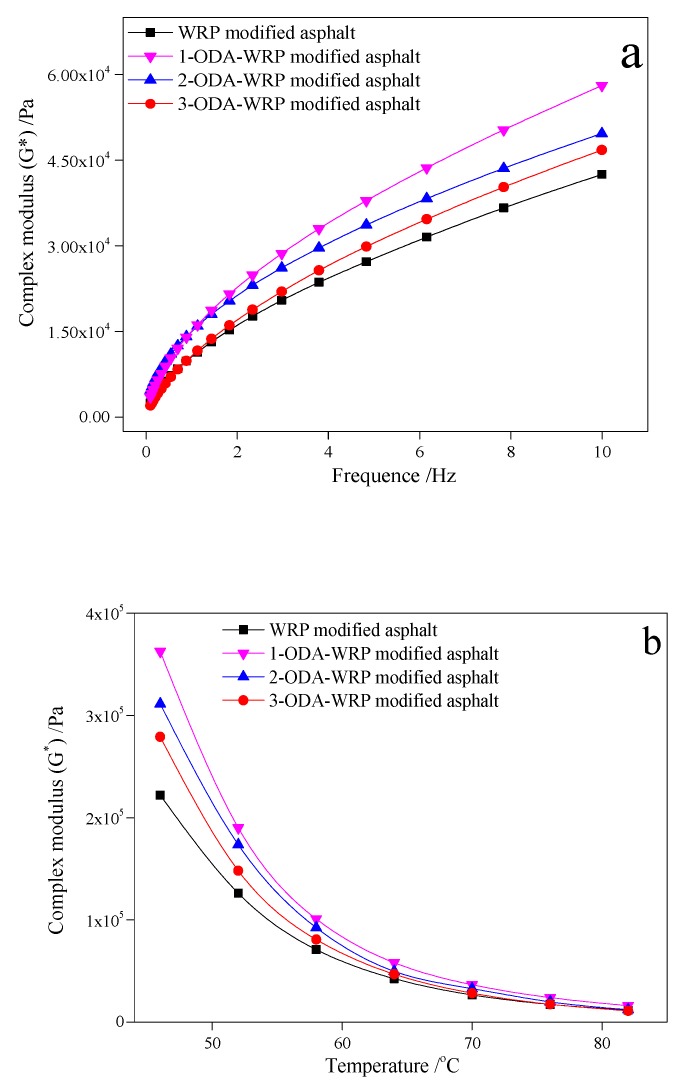
Variation in G* with: (**a**) frequency at a constant temperature of 64 °C and (**b**) temperature at constant frequency of 10 Hz for different kinds of ODA-WRP asphalt.

Figure 4a shows the variation in G* with frequency at a constant temperature and suggests that G* of each modified asphalt increased with the increase in frequency. Furthermore, G* of ODA-WRP-modified asphalt was larger than WRP-modified asphalt, while that of 1-ODA-WRP-modified asphalt was the largest (i.e., 5.81 KPa at 10 Hz, which was about 36.47% higher than that of WRP-modified asphalt). However, G* of 3-ODA-WRP-modified asphalt was smaller (4.68 KPa) corresponding to about a 10.12% increase as compared to that of WRP-modified asphalt. The variation in G* of ODA-WRP-modified asphalt with varying grafting amounts indicated that ODA could effectively improve the ability of asphalt to resist deformation, where a smaller amount of ODA grafted on WRP was more favorable [42]. A similar trend was also observed in Figure 4b. At a fixed shear frequency of 10 Hz, the G* of each modified asphalt gradually decreased with increasing temperature. However, the G* of ODA-WRP-modified asphalt always stayed higher than that of WRP-modified asphalt at all temperatures, while that of 1-ODA-WRP-modified asphalt was at maximum in all samples. It was also observed that with increasing the amount of ODA grafted on WRP, the value of G* gradually decreased.

#### 3.2.2. Storage Modulus (G′)

**Figure 5:**
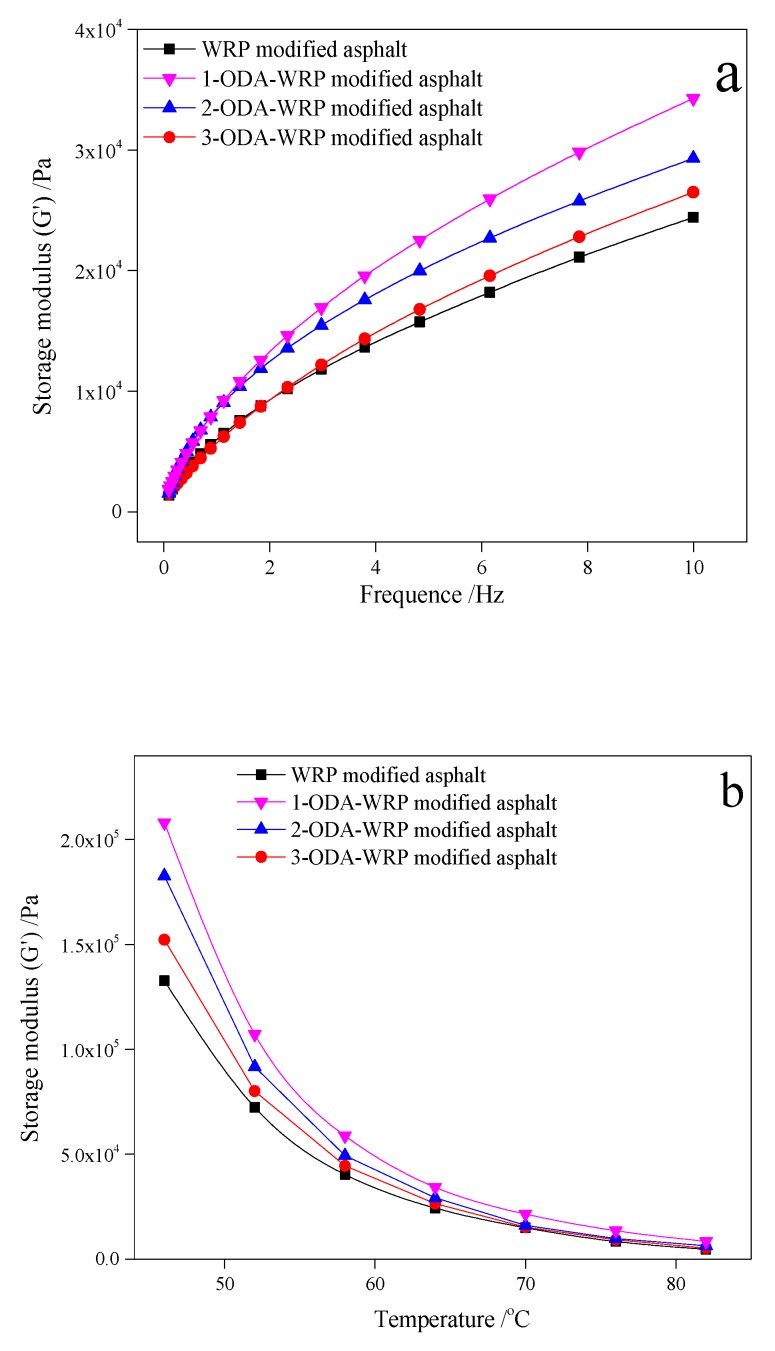
Variation in G′ with: (**a**) frequency at 64 °C and (**b**) temperature at 10 Hz for different kinds of ODA-WRP asphalt.

A similar trend to that of Figure 4 was observed in Figure 5, where the effect of frequency and temperature on G′ of the modified asphalt was compiled. At 64 °C (Figure 5a), the G′ of each group of asphalt increased with increasing frequency. The G′ of ODA-WRP-modified asphalt was larger than that of WRP modified asphalt, while that of 1-ODA-WRP-modified asphalt was the largest (3.43 KPa), equivalent to an increase of about 40.57% than WRP-modified asphalt at 10 Hz. The 3-ODA-WRP-modified asphalt had a smaller G′ (G′_10Hz_ = 2.93 KPa), which was about 20.08% higher than that of WRP-modified asphalt. At a fixed shear frequency of 10 Hz, the G′ of each modified asphalt gradually decreased with increasing temperature. However, G′ of ODA-WRP-modified asphalt was larger than that of WRP-modified asphalt at all temperatures, among which, that of 1-ODA-WRP-modified asphalt was the largest. Furthermore, with increasing the amount of ODA grafted on WRP, the value of G′ gradually decreased. Higher values of G′ suggested that the addition of ODA-WRP could improve the elastic properties of asphalt, which was related to the amount of ODA grafting content. In the test range of this study, increasing grafting amounts of ODA led to weaker effects of WRP on the elastic properties of SBS-modified asphalt.

#### 3.2.3. Loss Modulus (G″)

**Figure 6:**
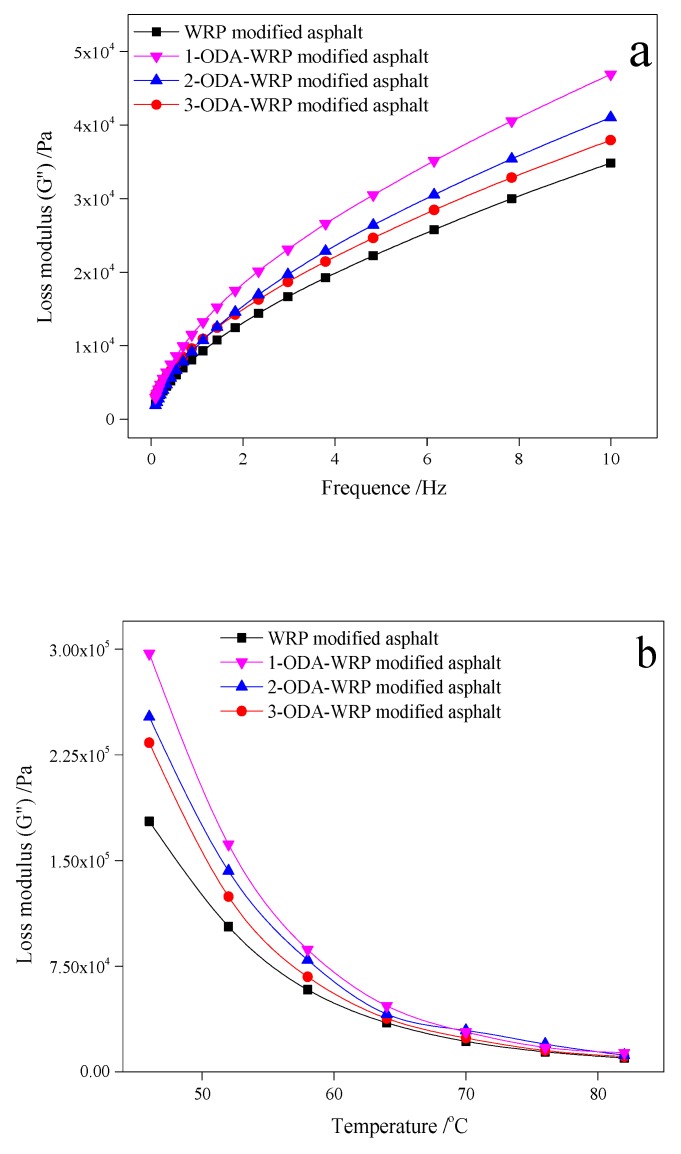
Variation in G″ with: (**a**) frequency at 64 °C and (**b**) temperature at 10 Hz for different kinds of ODA-WRP.

Figure 6 shows a similar trend of variation in G″, with frequency and temperature for different kinds of ODA-WRP, to that shown for G* and G′ in Figure 4 and Figure 5. In both Figure 6a,b, the G″ of ODA-WRP-modified asphalt was greater than that of WRP-modified asphalt. At 64 °C, 1-ODA-WRP-modified asphalt exhibited maximum G″ (i.e., 4.69 KPa), which was about 34.77% higher than that of WRP-modified asphalt. The increase in G″ indicated that the addition of ODA-WRP could improve the viscosity performance of asphalt, which was inversely proportional to the ODA content grafted on the WRP surface.

Based on the above results, one can conclude that the introduction of ODA onto the surface of WRP effectively improved the high temperature stability and viscoelastic properties of original asphalt. A generalized reason could be that the lipophilicity of ODA-WRP was higher than that of ordinary WRP, so it could firmly “catch” the surrounding asphalt molecules and the SBS molecular chain, thereby increasing its filling effect in the asphalt and the structural strength of the SBS network (to be discussed in FM analysis in Section 3.5.1). The decrease in the effect of ODA-WRP on asphalt performance with increasing ODA content could be related to the filling action of WRP in asphalt, as it could swell after absorbing the light components in asphalt [34]. However, with increasing ODA grafting content, the lipophilic groups on the surface of ODA-WRP were also increased, hence, increasing its interaction and affinity with the surrounding asphalt molecules. These excessive lipophilic groups caused the nearby heavy components to form a dense protective layer on the surface of WRP, thereby hindering the absorption of light components of asphalt by WRP and its own swelling. This was the main reason for the decreased performance of the high grafting amount of 3-ODA-WRP-modified asphalt than that of 1-ODA-WRP-modified asphalt [44]. The DSR results regarding the improvement in viscoelastic properties by ODA-WRPs (5%) SBS-modified asphalt were better than some reported studies that applied higher content of WRP or SBS [38,39,40], which makes the proposed study have greater economic value without compromising the mechanical properties of the final modified asphalt.

### 3.3. Multi-Stress Creep Recovery (MSCR) Test of Various Asphalts

Rutting factor (RF) is a physical quantity among asphalt’s rheological properties, which quantitatively and proportionally represents the ability of asphalt to resist high-temperature, permanent deformation. However, certain reports have shown that for polymer-modified asphalt (SBS, SBE, etc.), the value of the rutting factor does not represent the actual antirutting performance of asphalt [45]. AASHTO proposes that recovery rate (R) and the creep compliance (J_nr_) calculated by the MSCR test (via the same instrument used in the DSR test) can better determine the rutting resistance of polymer-modified asphalt [11,45]. However, the test sample needs to be subjected to short-term aging before test analysis.

According to AASHTO’s test protocol TP70-10, the high-temperature performance grade (PG) of each modified asphalt was measured under similar test conditions mentioned in Section 3.2. According to PG high-temperature grade measurement standards, the rutting factor (RF) (obtained from DSR test results) of the original asphalt and short-term aged asphalt should be greater than 1 KPa and 2.2 KPa, respectively, at the highest temperature [46]. Thus, RF for four types of modified asphalt were calculated and compiled in Table 2.

It can be seen from Table 2 that the RF values of various groups of asphalt reduced after short-term aging. This was because during the aging process, RP further absorbed light components in asphalt, and after reaching saturation, rupturing occurred, thereby forming a larger amount of small rubber particles. This resulted in decreasing the filling effect of WRP in asphalt, leading to a decrease in RF value.

**Figure 7:**
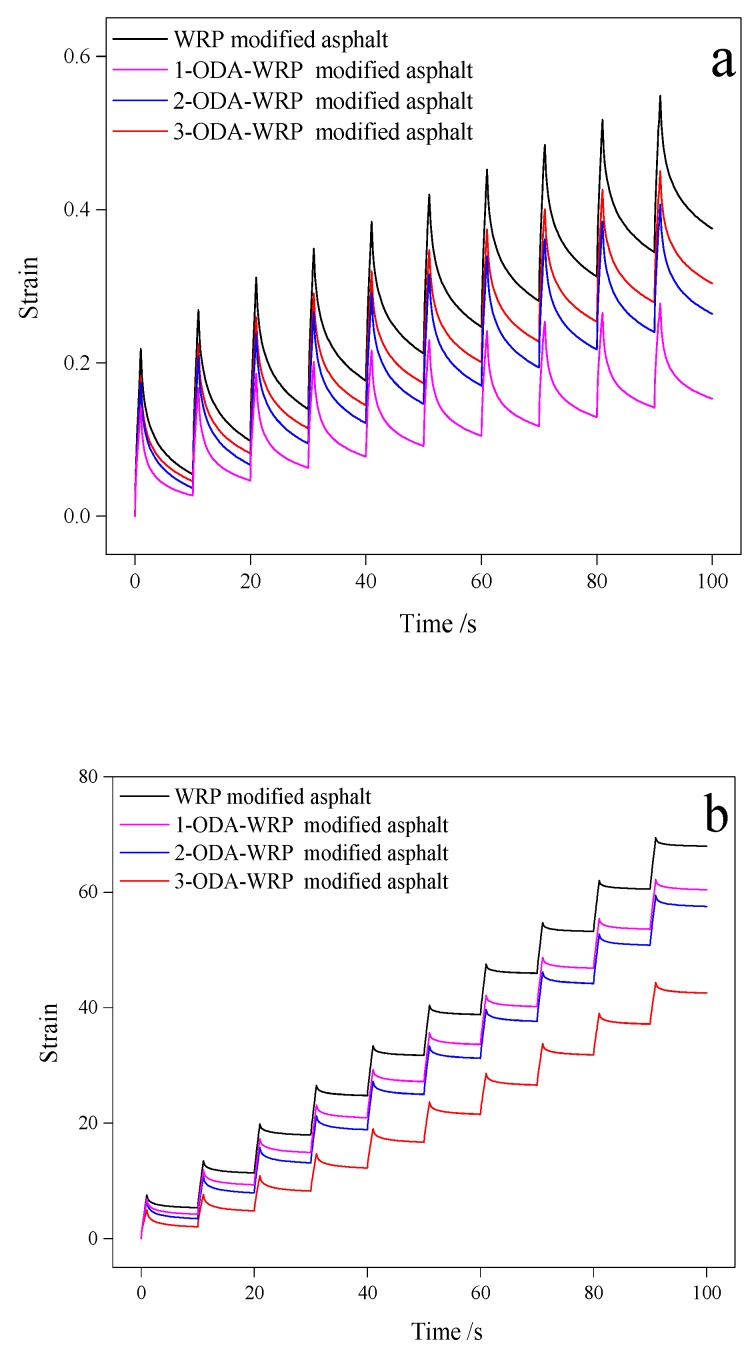
Time-strain relation of different asphalt samples at 82 °C under: (**a**) 0.1 KPa and (**b**) 3.2 KPa.

According to the PG grade measurement standard and the test results in Table 2, the PG high-temperature grade of WRP-modified asphalt and three kinds of ODA-WRP-modified asphalt belonged to PG86. Therefore, an MSCR test was performed at 82 °C. According to the test standards, the gap and the diameter for the test were 1 and 25 mm [45]. The MSCR test consisted of 1 s of creep loading followed by 9 s of recovery over multiple stress levels of 0.1 and 3.2 kPa at 10 cycles for each stress level. J_nr_ is the rutting potential index in the MSCR test, which is equal to the average non-recovered strain for the 10 creep and recovery cycles divided by the corresponding applied stress during those cycles. Through the test results, the time-strain diagram of each asphalt under the same stress was obtained and plotted in Figure 7.

Figure 7 shows that with increasing test time, the strain of the four modified asphalts also increased, suggesting the partial deformation of asphalt after 10 cycles of stress. In each stress cycle, consisting of a 1 s stress loading and 9 s unloading process, the four modified asphalts retained some of the strain. This increased the total strain of the asphalt with an increasing number of cycles and accumulating strains up to certain extent, which will eventually bring permanent damage to the asphalt. This situation was similar to the effect of external load on an asphalt road in daily life, and can simulate the rutting effect of the actual road surface to a certain extent. The increase in strain of asphalt under low stress (0.1 KPa), shown in Figure 7a, was smaller than that at higher stress (3.2 KPa), shown in Figure 7b, indicating greater deformation under higher stress. The strains of the three ODA-WRP-modified asphalts were smaller than those of WRP-modified asphalt, suggesting that the addition of ODA-WRP can effectively reduce the degree of deformation of asphalt under external forces. Among the three types of ODA-WRP-modified asphalt, 1-ODA-WRP- and 3-ODA-WRP-modified asphalts exhibited the smallest and largest strains, respectively.

Applying the MSCR test results of four types of asphalts, shown in Figure 7, combined with the corresponding action stress τ, the recovery rate R and the creep compliance J_nr_ were calculated by Equations (4) and (5), respectively [47,48]. R represents the extent to which the asphalt can recover after being subjected to an external force, and its value directly shows the recovery ability and anti-rutting performance of asphalt. J_nr_ represents the size of the unrecoverable part of asphalt accumulated by external forces. The smaller the value of J_nr_, the lower the permanent deformation of asphalt. The results of R and J_nr_ of the four types of modified asphalts under two different stresses are shown in Table 3.

(4) $$R=\frac{\gamma_{p}-\gamma_{nr}\;}{\gamma_{p}-\gamma_{0}},$$

(5) $$J_{nr}=\frac{\gamma_{nr}-\gamma_{0}\;}{\tau }.$$

Table 3 shows that when the stress was increased from 0.1 KPa to 3.2 KPa, the values of R of various modified asphalts were reduced, while J_nr_ values were improved to varying extents. This indicated that at higher applied stress, it was difficult for asphalt to return to its initial state, corresponding to a greater degree of permanent damage. Under low stress (0.1 KPa), the R of ODA-WRP-modified asphalt was higher than that of WRP-modified asphalt, indicating that the former can improve the anti-rutting performance of asphalt, while R of 1-ODA-WRP-modified asphalt was the largest (R_0.1_ = 0.890) among the three ODA-WRP-modified asphalts. Furthermore, the value of R of ODA-WRP-modified asphalt decreased with an increasing ODA grafting amount. Contrary to the change in R, J_nr_ values of the three ODA-WRP-modified asphalts were smaller than that of WRP-modified asphalt, indicating that the addition of ODA-WRP could effectively inhibit permanent damage to the asphalt caused by external forces. Among the various samples, J_nr_ of 1-ODA-WRP-modified asphalt was the smallest (J_nr0.1_ = 0.153 KPa^−1^), which was about 59.2% lower than that of WRP-modified asphalt (J_nr0.1_ = 0.375 KPa^−1^). A similar relation between R and J_nr_ was observed at higher stress (i.e., 3.2 KPa) for the four types of modified asphalts. Again, R of 1-ODA-WRP-modified asphalt was the largest, while J_nr_ was the smallest among the three ODA-WRP-modified asphalts (R_3.2_ = 0.365 and J_nr3.2_ = 1.328 KPa^−1^). The trend of R and J_nr_ indicated that the addition of ODA-WRP, under both stresses, could improve the anti-rutting performance of asphalt and reduce the permanent deformation of asphalt caused by external forces.

In order to simulate the anti-rutting performance of asphalt in a conventional climate, the four modified asphalts were tested at 64 °C by an MSCR test. Time-strain relation of each group of asphalt are shown in Figure S1. A similar trend to that in Figure 7 was observed for strain changes over time in Figure S1, though at significantly lower strain growth rates for the four modified asphalts at 64 °C. Lower temperatures increased the proportion of asphalt’s elastic properties and reduced the proportion of asphalt’s viscous properties [16]. The increase in the elastic properties of asphalt can dissipate the strain accumulated after many cycles of loading and unloading and, hence, it can increase the anti-rutting performance of asphalt. Similarly, identical trends to that in Figure 7, for R and J_nr_ for four types of modified asphalts at 64 °C, were observed as shown in Table S1. 

From MSCR test results at two temperatures, one can conclude that ODA-WRP can improve the anti-rutting performance and resistance to permanent deformation of asphalt compared to WRP. This was because lipophilic ODA-WRP could effectively improve the compatibility of WRP with asphalt, enabling WRP to “catch” nearby asphalt molecules and SBS molecular chains. This achieved a better filling effect and improved the strength of the SBS network in asphalt at certain loading amounts of ODA-WRP. Beyond a certain amount, the excessive lipophilic groups in ODA grafted over the WRP surface could cause adverse effects on both the recovery and anti-rutting performance of asphalt.

### 3.4. Stability of ODA-WRP in Asphalt

**Figure 8:**
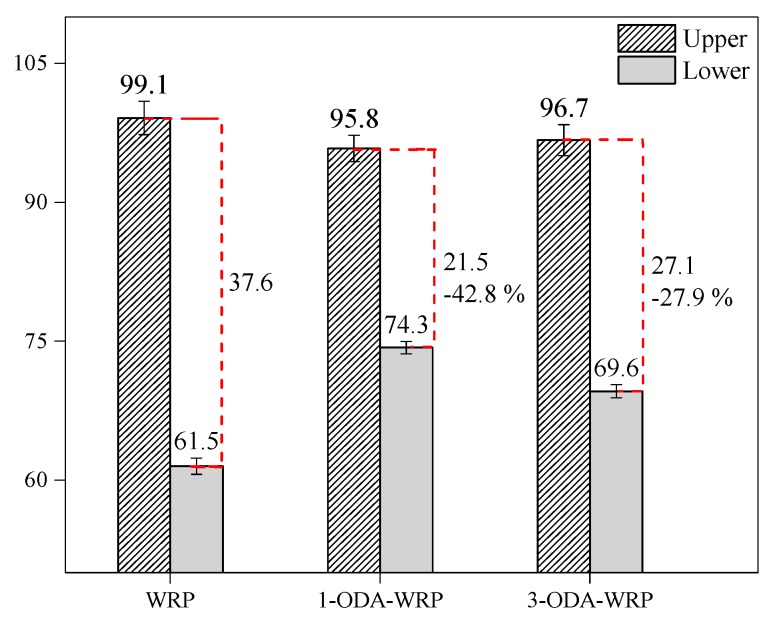
Segregation test results for various asphalt samples.

In order to better analyze the segregation of ODA-WRP in asphalt, the original WRP-modified asphalt, 1-ODA-WRP-modified asphalt, and 3-ODA-WRP were selected. The compatibility of WRP in asphalt was tested according to the test standards [35], and the results are shown in Figure 8.

It can be seen from Figure 8 that the softening point of WRP-modified asphalt had the largest difference (△T = 37.6 °C), suggesting a high degree of segregation [35]. On the contrary, 1-ODA-WRP-modified asphalt showed the smallest difference in the softening point (△T = 21.5 °C), which was about 42.8% lower than that of WRP-modified asphalt, while that of 3-ODA-WRP-modified asphalt was lower than WRP and higher than 1-ODA-WRP (△T = 27.1 °C). This showed that the separation of ODA-WRP-modified asphalt was lighter. The softening point of the upper part of the three groups of samples showed a significant increase compared with the original WRP-modified asphalt, and cross-linking phenomenon started to appear. This was because most of the rubber used to make automobile tires is vulcanized [20,21], hence, WRP contains a large amount of sulfur. After 48 h of high-temperature treatment, the WRP particles in the four modified asphalts became swollen and ruptured, thereby releasing a large amount of sulfur. Since the density of sulfur is lower, it floated on the upper asphalt, which resulting in the occurrence of cross-linking. At the same time, analyses of upper and lower parts of the same asphalt revealed a lower content of WRP in the upper part and a greater content in the lower part of asphalt. Compared to the original WRP/SBS-modified asphalt, the softening points of the upper part of the two types of ODA-WRP/SBS-modified asphalts were lower because of the lipophilic nature of ODA-WRP itself, which caused more ODA in the upper part of the sample.

### 3.5. Microscopic Analyses

#### 3.5.1. Fluorescence Microscopy (FM) Analysis of ODA-WRP in Asphalt

**Figure 9:**
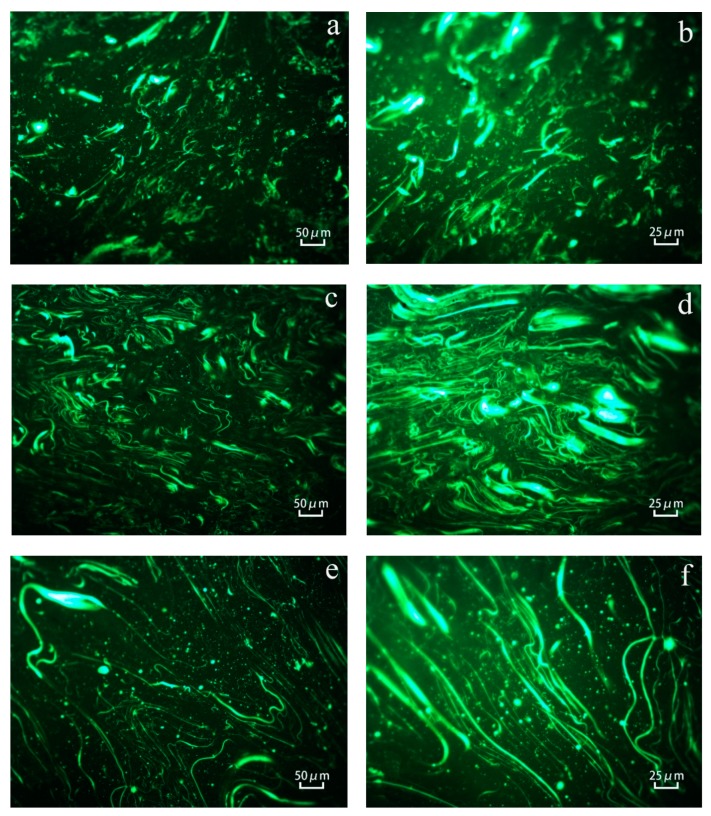
FM images of WRP at 200× (**a**) and 400× (**b**); 1-ODA-WRP at 200× (**c**) and 400× (**d**); and 3-ODA-WRP at 200× (**e**) and 400× (**f**).

In order to observe the distribution of WRP and modified ODA-WRP in asphalt, WRP-modified asphalt, 1-ODA-WRP-modified asphalt, and 3-ODA-WRP-modified asphalt were tested by FM. After heating the asphalt to a molten state, a small amount was dropped and flattened over a washed glass slide until the pitch was in the form of flakes. After cooling the sample to room temperature, FM analyses were performed. The results are shown in Figure 9, where the white light-emitting linear substance and bigger white light-emitting point-like substance is SBS, and the smaller white light-emitting point-like substance is WRP.

**Figure 10:**
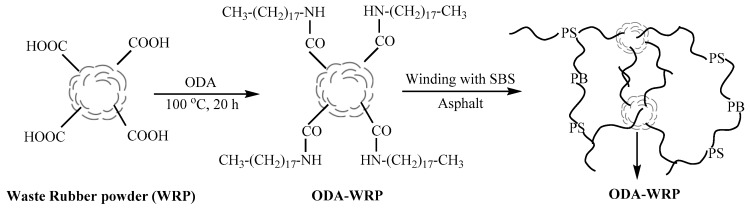
Formation mechanism of the ODA-WRP/SBS network structure.

**Figure 11:**
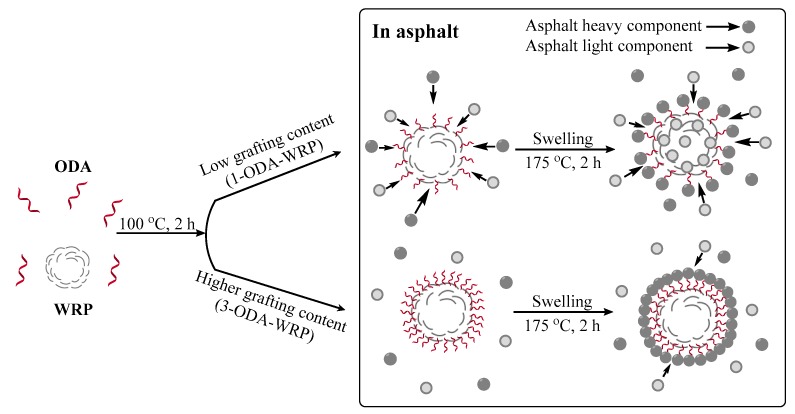
Swelling mechanism of different types of WRP.

Comparing the FM images in Figure 9a,c,e at a magnification of 200×, there are uniformly distributed WRP particles and a certain SBS network structure in the three modified asphalts. The density of the SBS network structure in ODA-WRP-modified asphalt was higher than that of WRP-modified asphalt, while that in 1-ODA-WRP-modified asphalt was greater than that in 3-ODA-WRP-modified asphalt. This was because the ODA lipophilic group that was on the WRP surface was a long-chain alkane, which was intertwined with an SBS molecular chain in asphalt, thus promoting the formation of an SBS network structure (Figure 10). However, grafting WRP with higher amounts of ODA will promote the heavy component of asphalt to encapsulate the particles. This will reduce the contact chances of ODA-WRP and SBS molecules, and will, thus, decrease the promotion effect on the SBS network structure, though it is still higher than WRP-modified asphalt. FM images of the three kinds of asphalt at 400× magnification revealed that WRP had the smallest particle size and highest density in 1-ODA-WRP, and it had the largest particles and lowest density in 3-ODA-WRP-modified asphalts. The change in particle size of WRP proved that grafted ODA on the surface of WRP could effectively improve the swelling and rupturing rate of WRP in asphalt. Higher amounts of ODA led to a slower swelling and rupturing rate of WRP, because WRP grafted with a higher amount of ODA caused the heavy components of asphalt to wrap the particles, avoiding absorbing the light components of asphalt by WRP, as shown in Figure 11.

#### 3.5.2. Structural and Textural Characterization of ODA-WRP

**Figure 12:**
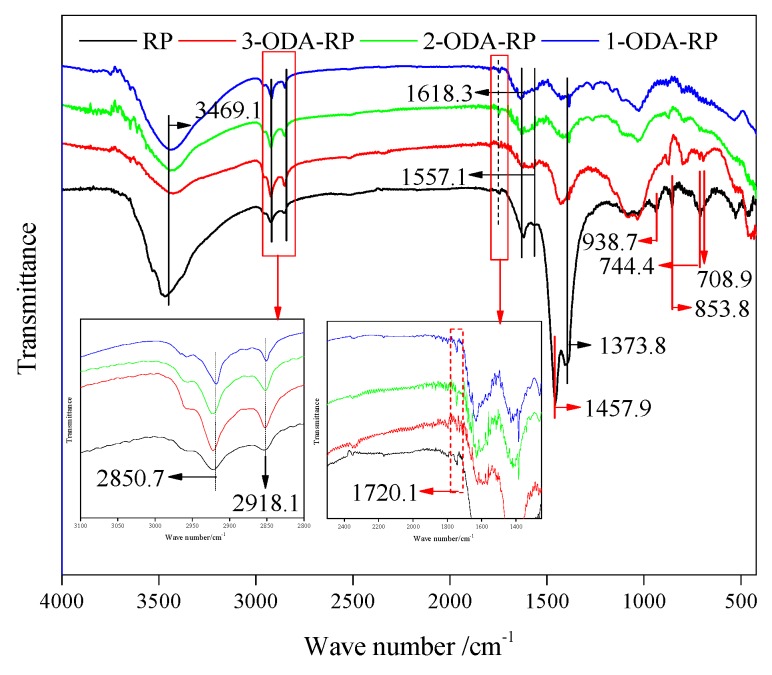
FT-IR spectra of WRP, 1-ODA-WRP, 2-ODA-WRP, and 3-ODA-WRP.

Figure 12 and Table 4 show the FT-IR spectra of WRP, 1-ODA-WRP, 2-ODA-WRP, and 3-ODA-WRP. The characteristic peaks in WRP appearing at 2918.1 cm^−1^ and 2850.7 cm^−1^ were the stretching vibration peaks of methyl (–CH_3_) and methylene (–CH_2_–). The peaks appearing at 1457.9 cm^−1^ and 853.8 cm^−1^ were ascribed to the characteristic bands of natural rubber (NR) [49]. The peaks appearing at 744.4 cm^−1^ and 708.9 cm^−1^ were the characteristic bands of monosubstituted benzene [50], while those at 938.7 cm^−1^ were the characteristic bands of trans-unsaturated C–H out-of-plane bending vibrations, representing styrene-butadiene rubber (SBR) [51]. These results suggested that WRP used in this experiment was mainly composed of NR, butadiene rubber, and SBR. 

WRP also exhibited the characteristic peaks of two oxidizing groups: a hydroxyl group (C–OH, 3469.1 cm^−1^ and 1373.8 cm^−1^) [17,34] and a carboxyl group (–COOH, 1720.1 cm^−1^) [55,56,57,58]. Among these, the lower content of –COOH was in line with earlier reports [32,59,60]. Comparing the FT-IR spectra of ODA-WRP and WRP, it can be found that the characteristic peaks of –NHCO– (1618.3 cm^−1^ and 1557 cm^−1^) [34] existed in ODA-WRP and WRP, so ODA-WRP could not be determined by the change of the amide bond. Moreover, the characteristic peak of –COOH in WRP after modification slightly decreased, but the generation of ODA-WRP could not be proved because of the small change in –COOH [34]. However, the characteristic peaks of –CH_3_ and –CH_2_– at 2918.1 cm^−1^ and 2850.7 cm^−1^, respectively, significantly increased after WRP modification, and the intensity of the characteristic peaks gradually increased with an increasing ODA grafting amount. This indicated that more chain alkanes were introduced in the modified WRP, which was derived from the lipophilic group moiety –(CH_2_)_17_CH_3_ of ODA. In summary, the FT-IR test results could partially prove the successful grafting of ODA on WRP.

**Figure 13:**
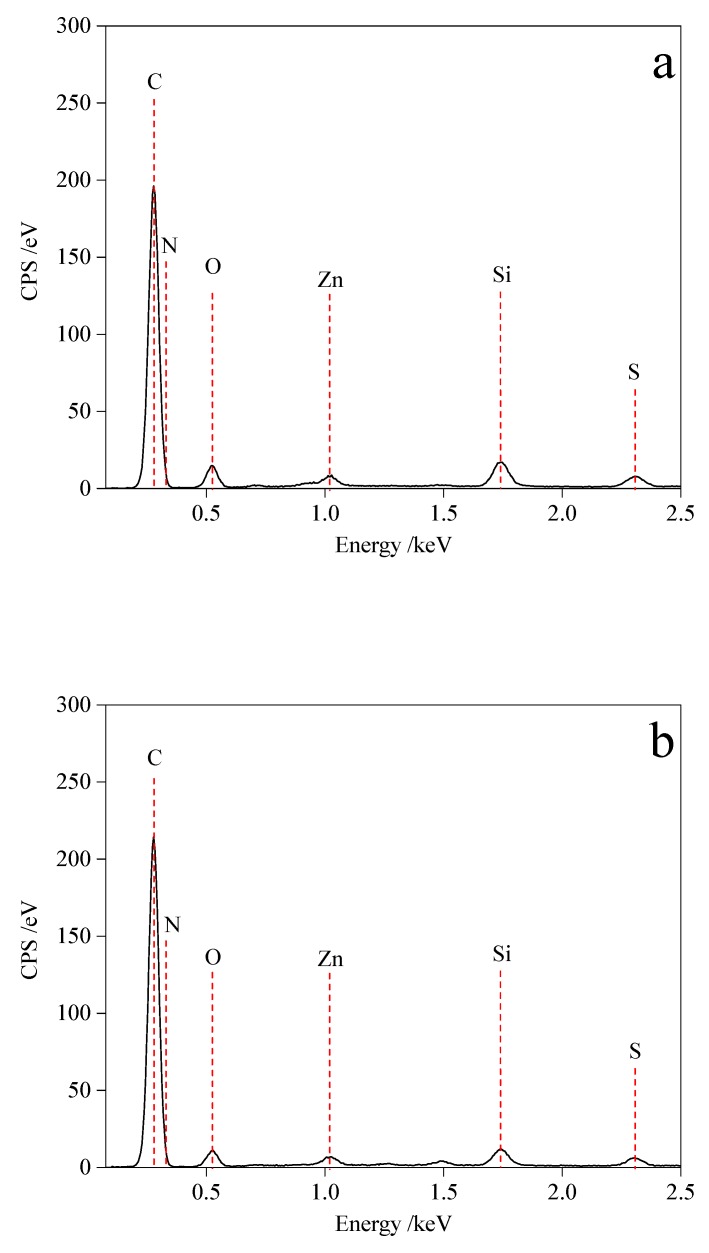

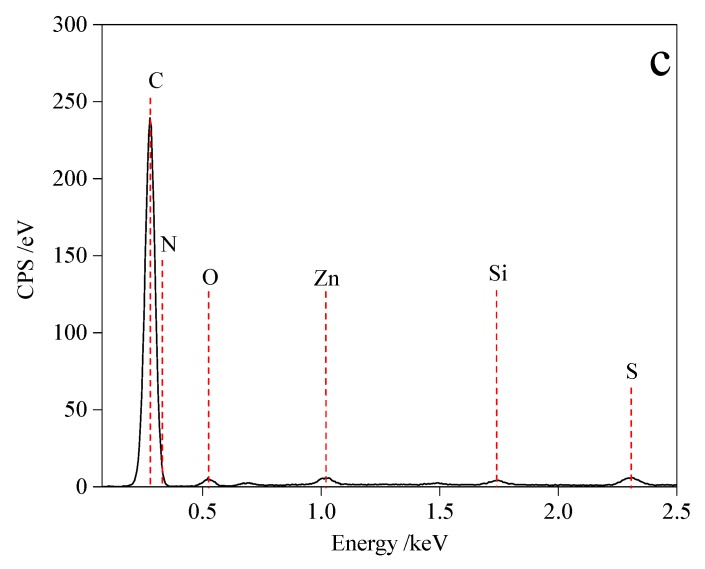
The energy dispersive spectroscopy (EDS) analyses of (**a**) WRP, (**b**) 1-ODA-WRP, and (**c**) 3-ODA-WRP.

Figure 13a–c and Table 5 show the EDS test results of WRP, 1-ODA-WRP, and 3-ODA-WRP and the proportion of atoms of various elements, which suggest that WRP contained C, N, O, Zn, and Si elements. The presence of Zn and Si may arise from grinding machine wear during waste rubber grinding. Because of the close EDS energy values of C and N, their peaks greatly coincided. Comparison of the EDS results of three types WRP samples revealed that ODA grafted on the surface of WRP led to the increase in C content, while the O and N content decreased. Additionally, as the amount of ODA grafting increased, the magnitude of change of the three types elements increased. This could be because an ODA molecule, upon reacting with WRP, will result in the conversion of –COOH and –NH_2_ to –NHCO–, consuming one –OH group, resulting in a decrease in O content. At the same time, an 18 carbon oleophilic group (–(CH_2_)_17_CH_3_) was grafted onto the WRP surface, which caused a substantial increase in C content. Although a single atom can be introduced into the WRP under a single reaction, the number of C atoms excessively increased, thus the ratio of N atoms was lowered. Changes in the contents of C, O, and N demonstrated the successful synthesis of ODA-WRP. By comparing the relative growth of 1-ODA-WRP and 3-ODA-WRP with WRP, it can be seen that as the ratio of ODA increased, the content of ODA that can react with WRP decreased. This was also because of the small content of –COOH in WRP.

**Figure 14:**
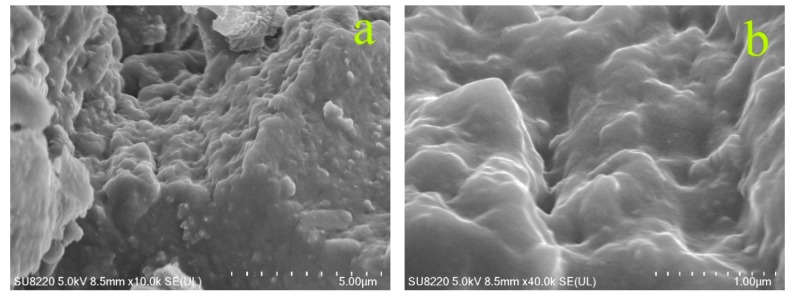

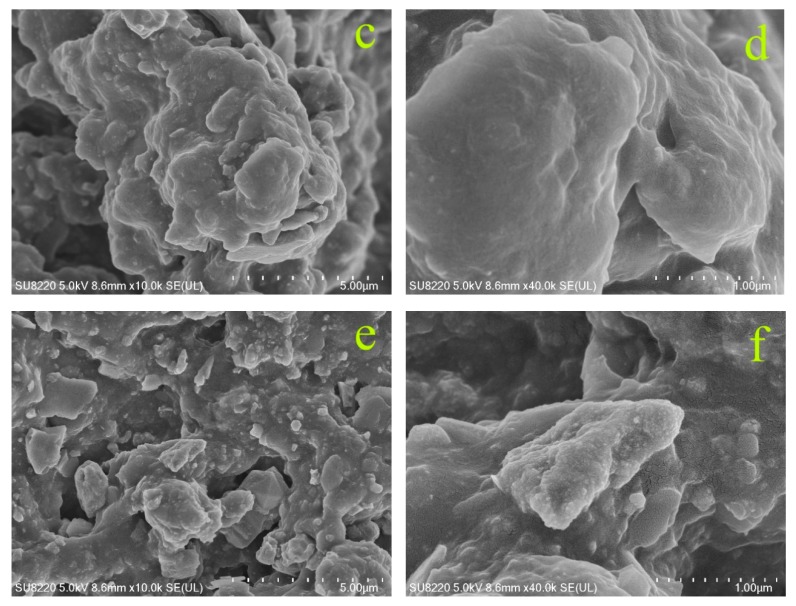
SEM analysis of WRP (**a**,**b**), 1-ODA-WRP (**c**,**d**), and 3-ODA-WRP (**e**,**f**).

SEM images in Figure 14a–f corresponding to WRP, 1-ODA-WRP, and 3-ODA-WRP revealed that unmodified WRP (Figure 14a,b) had an irregular, uneven surface with a large number of holes. In modified WRP (Figure 14c–f), the holes still exhibited an irregular black structure, but the original holes were reduced, the appearance of undulation was reduced, and the surface became rougher, which was accredited to the grafting of ODA on the WRP surface. Comparing the SEM images of the two kinds of ODA-WRP, the surface of 3-ODA-WRP (Figure 14e,f) with higher ODA grafting amounts was much rougher with a greater degree of undulation than that of 1-ODA-WRP (Figure 14c,d).

## 4. Conclusions

In this study, ODA was grafted onto a WRP surface using a covalent bonding grafting approach. Three types of ODA-WRP with different ODA grafting amounts were prepared and used as modifiers to synthesize ODA-WRP-SBS-modified asphalt. Data from three major indicators showed that ODA-WRP incorporation can effectively improve the plasticity, stability, and viscosity of SBS-modified asphalt. DSR and MSCR test results showed that the addition of ODA-WRP can effectively improve the high temperature stability, viscoelasticity, and rutting resistance of SBS-modified asphalt. Segregation tests revealed improved compatibility of modified ODA-WRP in SBS-modified asphalt. The modification effect of ODA-WRP on SBS-modified asphalt decreased with an increasing ODA grafting amount on WRP because of the presence of more lipophilic long-chain alkyl groups on the ODA-WRP surface, which allowed the WRP to “catch” the nearby asphalt components and SBS molecular chains. WRP enhanced the development of the SBS network, which led to superior mechanical properties of 1-ODA-WRP-modified asphalt, as confirmed by FM analysis. FT-IR and SEM results demonstrated the successful grafting of ODA onto the WRP surface. The design of ODA-WRP in this study highlights a simplified preparation, significant enhancement in mechanical properties, and an environmentally friendly process, which can be applied in the preparation of modified asphalt in construction and highway applications.

## Figures and Tables

**Table 1:** Modified asphalt composition and terminology of each group having 5% WRP content.

Type of WRP	Simplified Name
WRP	WRP-modified asphalt
ODA-WRP (0.5:20)	1-ODA-WRP-modified asphalt
ODA-WRP (1:20)	2-ODA-WRP-modified asphalt
ODA-WRP (2:20)	3-ODA-WRP-modified asphalt

**Table 2:** Rutting factor (RF) value of different original and short-term aged asphalts at different temperatures.

Temperature (°C)	RF/KPa			
	WRP-Modified Asphalt	1-ODA-WRP-Modified Asphalt	2-ODA-WRP-Modified Asphalt	3-ODA-WRP-Modified Asphalt
70	8.55	14.61	10.64	9.72
76	5.16	8.74	6.59	5.37
82	2.91	5.27	4.14	3.39
Rotating film oven test (RTFOT) aging treatment				
82	2.64	3.61	3.22	3.05

**Table 3:** R and J_nr_ of various samples at 0.1 KPa and 3.2 KPa (82 °C).

Samples	R and J_nr_ values			
	R_0.1_	J_nr0.1_/KPa^−1^	R_3.2_	J_nr3.2_/KPa^−1^
WRP	0.821	0.375	0.203	2.124
1-ODA-WRP	0.890	0.153	0.365	1.328
2-ODA-WRP	0.845	0.264	0.292	1.798
3-ODA-WRP	0.827	0.304	0.264	1.888

**Table 4:** FT-IR peaks and representative functional group (ν: stretching, β: in-plane bending, γ: out-of-plane bending, ω: wagging, ρ: rocking, δ: deformation, and (a)sym: (a)symmetric.).

Number	FTIR Peak Position/cm^−1^	Representative Composition	References
1	708.9	ν C-H (benzene)	[52]
2	744.4	ρ –CH_2_–	[53]
3	853.8	γ C=C–H (cis-1,4 addition)	[53,54,55,56]
4	938.7	ν C–C	[53]
5	1373.8	δ (a)sym. –CH3	[53,55,56,57]
6	1457.9	δ –CH_2_– + ρ –CH_3_	[53,55,57]
7	1557.1	Amide II: β N–H + ν C–N	[57]
8	1618.3	Amide I: ν R1–(C=O)–NH–R2	[57]
9	1720.1	ν R_1_–(C=O)–OH	[54,56,57]
10	2850.7	ν sym. –CH_2_–	[53,55]
11	2918.1	ν sym. –CH_3_	[53,55]
12	3469.1	ν –OH	[17,34]

**Table 5:** Atomic number proportion of each element under the EDS test.

Name	Element		
	C (at %)	N (at %)	O (at %)
WRP	86.15	1.69	12.16
1-ODA-WRP	89.78	1.62	8.60
3-ODA-WRP	93.83	1.48	4.69

## Supplementary Materials

The following are available online at https://www.mdpi.com/2073-4360/11/4/665/s1, Figure S1. Time-strain relation of different asphalt samples at 64 °C under: (a) 0.1 KPa and (b) 3.2 KPa, Table S1. R and Jnr of various samples under 0.1 KPa and 3.2 KPa at 64 °C.
