# Supplementary material for: Preparation of Octadecyl Amine Grafted over Waste Rubber Powder (ODA-WRP) and Properties of Its Incorporation in SBS-Modified Asphalt
Source: Polymers (Basel). 2019 Apr 11;11(4):665. doi: 10.3390/polym11040665 (PMC6524018; doi:10.3390/polym11040665)
Supplement: Supplementary file 1 [file polymers-11-00665-s001.pdf]

Supporting Information

Preparation of Octadecyl Amine Grafted over Waste Rubber Powder (ODA-WRP) and Properties of Its Incorporation in SBS-Modified Asphalt

Meizhao Han[1], Xiang Zeng[1], Yaseen Muhammad[2,3], Jing Li[1,2], Jing Yang[1], Song Yang[1], Yunhao Wei[1], Fei Meng[1]*

[1]School of Chemistry and Chemical Engineering, Guangxi University, Nanning 530004, China

[2]Guangxi Key Laboratory of Petrochemical Resource Processing and Process Intensification Technology, Guangxi University, Nanning 530004, China

[3]Insitute of Chemical Sciences, University of Peshawar, 25120, KP, Pakistan

Corresponding author: Jing Li
Email: lijingsophia1234@163.com

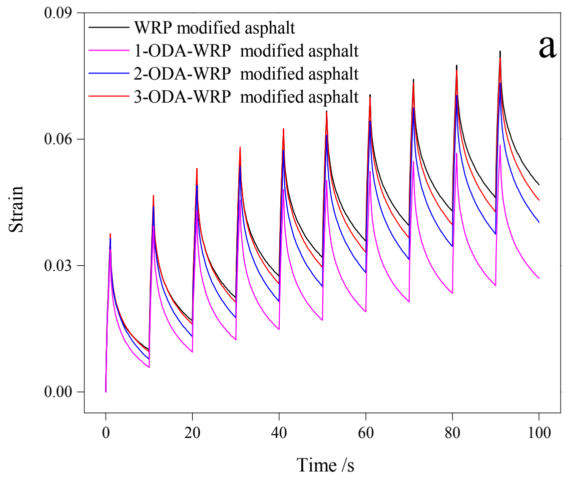

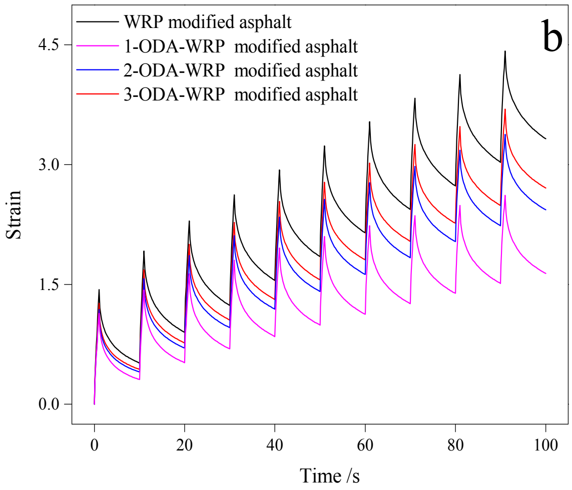

Fig. S1. Time-strain relation of different asphalt samples at 64 °C under: (a) 0.1 KPa and (b) 3.2 KPa

Table S1. R and J_{nr} of various samples under 0.1 KPa and 3.2 KPa at 64 °C

Samples	Properties			
	$R_{0.1}$	$J_{nr0.1}$/KPa^{-1}	$R_{3.2}$	$J_{nr3.2}$/KPa^{-1}
WRP	0.860	0.049	0.762	0.104
1-ODA-WRP	0.919	0.027	0.853	0.051
2-ODA-WRP	0.888	0.040	0.790	0.076
3-ODA-WRP	0.877	0.046	0.780	0.085
